# Pathogenicity and virulence of *Mycobacterium leprae*

**DOI:** 10.1080/21505594.2022.2141987

**Published:** 2022-11-03

**Authors:** Mariko Sugawara-Mikami, Kazunari Tanigawa, Akira Kawashima, Mitsuo Kiriya, Yasuhiro Nakamura, Yoko Fujiwara, Koichi Suzuki

**Affiliations:** a Department of Clinical Laboratory Science, Faculty of Medical Technology, Teikyo University, Tokyo, Japan; b West Yokohama Sugawara Dermatology Clinic, Yokohama, Japan; c Department of Molecular Pharmaceutics, Faculty of Pharma-Science, Teikyo University, Tokyo, Japan

**Keywords:** Leprosy, *Mycobacterium leprae*, macrophage, Schwann cell, pseudogene, lipids metabolism

## Abstract

Leprosy is caused by *Mycobacterium leprae* (*M*. *leprae*) and *M. lepromatosis*, an obligate intracellular organism, and over 200,000 new cases occur every year. *M. leprae* parasitizes histiocytes (skin macrophages) and Schwann cells in the peripheral nerves. Although leprosy can be treated by multidrug therapy, some patients relapse or have a prolonged clinical course and/or experience leprosy reaction. These varying outcomes depend on host factors such as immune responses against bacterial components that determine a range of symptoms. To understand these host responses, knowledge of the mechanisms by which *M. leprae* parasitizes host cells is important. This article describes the characteristics of leprosy through bacteriology, genetics, epidemiology, immunology, animal models, routes of infection, and clinical findings. It also discusses recent diagnostic methods, treatment, and measures according to the World Health Organization (WHO), including prevention. Recently, the antibacterial activities of anti-hyperlipidaemia agents against other pathogens, such as *M. tuberculosis* and *Staphylococcus aureus* have been investigated. Our laboratory has been focused on the metabolism of lipids which constitute the cell wall of *M. leprae*. Our findings may be useful for the development of future treatments.

## GRAPHICAL ABSTRACT

**Figure:**
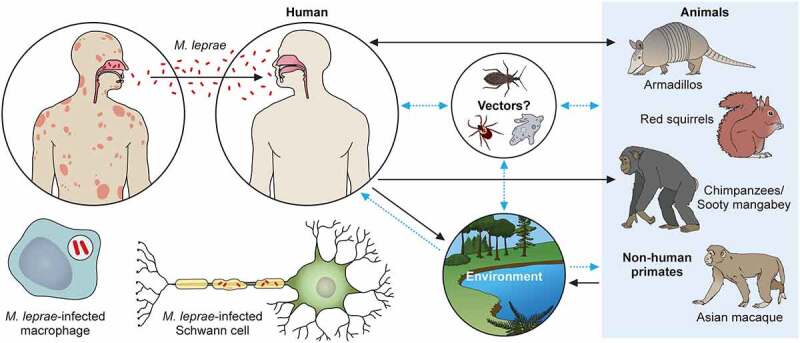

## Introduction

Leprosy is caused by *Mycobacterium leprae* (*M*. *leprae*), which was discovered by Gerhard Armauer Hansen of Norway in 1873 [1,2] and *M. lepromatosis* [3]. Leprosy is a chronic infectious disease which occurs worldwide. Globally, about 80% of newly registered cases are found in Brazil, India and Indonesia [4]. Areas having high endemicity are found within countries at a sub-district level [5]. New leprosy cases have been remarkably reduced by multidrug therapy (MDT) developed with the support of the World Health Organization (WHO) [6], but in 2019 around 200,000 cases were still reported from over 100 countries [4]. The WHO has designated leprosy as a neglected tropical disease (NTD) [7]. NTDs are infectious diseases that are targeted for eradication under the Sustainable Development Goals (SDGs), which are positioned as universal goals of the WHO [8]. In addition, the Global Leprosy Strategy for the years 2021–2030 entitled “Towards zero leprosy” has begun [9]. The goals of this strategy include: (a) no new autochthonous cases in 120 countries, (b) a 70% reduction of annual new cases, (c) a 90% reduction in the incidence of new Grade-2 disability (G2D) cases, and (d) a 90% decrease in the incidence of paediatric leprosy cases [9].

*M.leprae* is an obligate intracellular organism. This pathogen affects mostly the skin and the peripheral nerves [10]. This bacterium preferentially invades dermal histiocytes (tissue macrophages) and Schwann cells in peripheral nerves [11]. Skin lesions occur as pale papules or rashes with erythematous infiltration. Leprosy has been classified into five types using the Ridley-Jopling classification: tuberculoid (TT), borderline tuberculoid (BT), mid-borderline (BB), borderline lepromatous (BL) and lepromatous (LL). TT is associated with strong cellular immunity and low humoral immunity with granulomatous local skin lesions, whereas LL is characterized by strong humoral immunity. Nerve damage affected by this bacillus induces neuropathy with sensory and motor neuronal impairment. Moreover, the leprosy reaction is an intense immune reaction of the host against *M. leprae*. It is also a major factor that leads to disabling neurological disorders.

Delays in diagnosis and inadequate treatment of leprosy are responsible for a large variety of clinical symptoms. These symptoms frequently cause deformity and disability in perpetuity, often resulting in stigma. In particular, sensory nerve damage results in numbness and analgesia that can cause repeated injuries and subsequent loss of limbs. Motor neuropathy accompanying peripheral neuropathy causes major problems in activities of daily living (ADL) such as hand movement and gait, whereas secondary disuse muscle atrophy further impairs ADL to create a vicious cycle of disease.

A leprosy control program led by the WHO focused on early detection of patients and early treatment with antimicrobial agents to control new cases globally [12]. However, no effective treatment for peripheral neuropathy has yet been developed, and even after MDT patients may still experience serious disability. The WHO held informal consultations on monitoring the rate of G2D and the applicability of chemoprophylaxis to develop measures to improve leprosy [13]. WHO data indicate that there are over 10,000 newly registered G2D cases annually; more than 90% of these registrations belong to global priority countries over the past 10 years. Even though leprosy is now understood to be a controllable disease with MDT, the need to treat leprosy reactions and relapse remains. There is also a significant need for long-term treatment, and there is concern about the emergence of MDT-resistant strains [14].

The biological characteristics of *M. leprae* may contribute to clinical manifestations as well as challenges in treatment for leprosy. During infection, *M. leprae* parasitizes host cells and modifies the host cellular environment to promote its survival. Cell wall components of the bacilli may be important factors for such parasitization, since cell wall components not only elicit host immunity, but can also act as signalling factors that aid its survival [15]. *M. leprae* has many pseudogenes and is therefore only capable of surviving in host cells, using the functions of host cells for its survival. Further, *M. leprae* has been shown to survive in soil for more than 46 days [16]. Although many genes required for *M. leprae* infection, colonization and growth are preserved, many other genes have been discarded. Thus, *M. leprae* carries out various metabolic mechanisms, including lipid metabolism essential for cell wall synthesis, by parasitizing host cell machinery. Survival of the bacteria while immunologically concealed within host cells may require evasion of immune surveillance mechanisms to allow long-term parasitization. In this review, we summarize the pathogenicity of *M. leprae*, with particular focus on the underlying molecular mechanisms that enable intracellular parasitization.

## Pathogenicity and infection of *M. leprae*

### Bacteriology

*M.leprae* is an obligate intracellular organism and the taxonomic classification of this bacillus comprises the class Schizomycetes, order Actinomycetales, family Mycobacteriaceae, and genus *Mycobacterium* [17,18]. The bacteria are slightly curved, and measure 1–8 μm in length and 0.3–0.5 μm in diameter. *M. leprae* is an acid-alcohol-fast bacillus, non-motile and microaerophilic. *M. leprae* mainly infects and invades skin macrophages and Schwann cells in the peripheral nerves to produce a chronic infection in humans. Using the Gram strain, *M. leprae* tests as invisible, producing negatively stained representations known as “ghosts,” or as rod-shaped Gram-positive bacilli [19,20]. Due to its higher lipid content, *M. leprae* does not become discoloured by acid-alcohol with the Ziehl-Neelsen stain, a red stain that contains fuchsin. Thus, it appears as characteristic acid-alcohol-resistant bacilli using a slit skin smear test [19,20] (Figure 4a). *M. leprae* multiplies very slowly, requiring 12 to 14 days for generation, compared to the longer time (20 hours) needed by *Mycobacterium tuberculosis* (*M. tuberculosis)* [21,22]. Although 37 °C is the standard incubation temperature used for most pathogens, *M. leprae* requires a low temperature for growth [23–25]. Therefore, in humans it tends to preferentially parasitize cooler areas such as the skin, nasal mucosa and ears. *M. leprae* was reported to survive up to 46 days in the environment [16,24–26].

### Bacterial components

**Figure 1.**
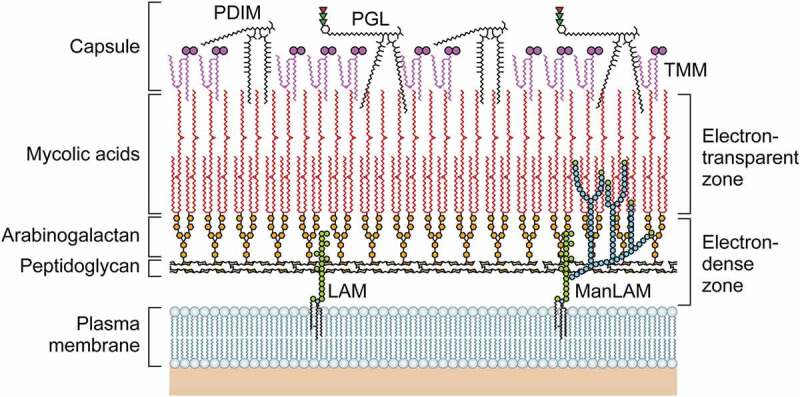
The structure of the *M. leprae* cell wall. The *M. leprae* cell wall consists of an inner and outer layer that surround a plasma membrane. The outermost layer includes PGLs that compose capsules. The electron-dense inner layer of cell wall contains PGN, AG, and mycolic acids. The outer cell wall, which is an electron-dense layer, consists of lipid-linked polysaccharides such as LAM, LM, and phthiocerol-containing lipids including phthiocerol dimycocerosate and dimycolyl trehalose. Mycolic acids link to arabinan chain termini and compose the inner leaflet of a pseudo lipid bilayer. An outer leaflet contains TMM mycolic acids and PDIM and PGL mycocerosoic acids. Small amounts of TMM also exist in the cell wall of *M. leprae*.

*M.leprae* has a thick cell wall surrounding the plasma membrane that comprises inner and outer layers [27] (Figure 1). The outermost layer includes the phenolic glycolipids (PGLs) that compose the capsules [28] and that contain a range of lipids, with phthiocerol dimycocerosate (PDIM) and PGL-I predominating [29]. The innermost layer beyond the plasma membrane is rigid and electron-dense, consisting of peptidoglycan (PGN), arabinogalactan (AG), and mycolic acids. The outer cell wall also contains lipid-linked polysaccharides such as lipomannan (LM), lipoarabinomannan (LAM), phthiocerol-containing lipids (e.g. PDIM), and dimycolyl trehalose [30,31]. The inner leaflet of the pseudo-lipid bilayer consists of linked mycolic acids and arabinan chain termini. An outer leaflet is composed of PGLs, mycolic acids with trehalose mono-mycolate (TMM) and mycocerosoic acids of PDIM. The *M. leprae* cell wall includes small amounts of TMM [32]. The *M. leprae* cell wall includes more mycolic acid than that of *M. tuberculosis* (the ratio of mycolic acid to PGN is 21:10 versus 16:10).

PGL-I containing trisaccharides are located at the outermost surface and interact with the host cell membrane [33]. The PGL-I triglycosyl unit is composed of phenol-PDIM and the specific trisaccharide of *M. leprae* (3,6-di-*O-*methylglucose linked α-1→4 to 2,3-di-*O-*methylrhamnose linked β-1→2 to 3-*O-*methylrhamnose) in a glycosidic bond to the constituent phenol [34]. PGL-I is thought to be involved in host-pathogen interactions, including the suppression of inflammatory cytokine secretion [35,36]. The adhesion and invasion of Schwann cells by *M. leprae* promotes neural damage [37,38] by increasing inducible nitric oxide synthase (iNOS) in the host macrophages [36]. In LL, there are high antibody titres against natural synthetic trisaccharide and disaccharide representing the PGL-I triglycosyl unit [39–41]. Thus, PGL-I has proven useful for the serodiagnosis of this disease.

LAM consists of three constituents: a mannosyl-phosphatidyl-myo-inositol (MPI) anchor, a polysaccharide backbone, and capping moieties [42]. Three types of LAM are known and these can be differentiated by structure: mannose-capped LAM (ManLAM), phospho-myo-inositol-capped LAM (PILAM) and non-capped LAM (AraLAM). These structural variants show different immunomodulatory properties, e.g. LAMs are capped by ManLAM in *M. leprae*. The immunomodulatory roles of LAM are suppression of *T*-cell activation [43], interferon (IFN)-γ-mediated induction of macrophage gene expression [44], inhibition of protein kinase C activity, scavenging of oxygen radicals [45], and induction of macrophage-associated cytokines such as tumour necrosis factor-α (TNF-α) [46,47].

*M*. *leprae* PGN belongs to the chemotype IV group and contains peptide side chains containing diaminopimelic acid (DAP) [48,49]. According to previous analyses of the *Mycobacterium* PGN structure, several compositions were identified [50–52]. These include *N*-glycolylmuramic acid (MurNGlyc), cross-links of DAP-DAP, and modifications of the carboxylic acid functional groups of DAP and D-Glu. A number of these attributes are related to PGN of *M*. *leprae* [53–56], which was previously reported to comprise MurNGlyc based on estimates of the glycolic acid content [57]. However, MurNAc is the only type of muramic acid that is evident in *M. leprae* PG [55]. An immune response is initiated by the nucleotide-binding oligomerization domain (NOD)-like receptors (NLRs) upon recognition of the bacterial PGN, with a muramyl tripeptide containing meso-DAP being the smallest PGN recognizable by NOD1 [58]. Furthermore, muramyl peptides which include amidated meso-DAP are bound less strongly by NOD1 [58,59]. When the L-Ala of the muramyl dipeptide was replaced with D-Ala, the stimulation of NOD2 activity was eliminated [60]. Since *M. leprae* PGN contains amidated DAP and Gly residues, it may evade host innate immune responses mediated by NOD1 and NOD2 [55].

### Pseudogenes

As mentioned above, the loss of many functional genes from the *M. leprae* genome requires it to parasitize host cells for survival. 2001 saw the complete genome sequencing of *M. leprae*. The 3.3 Mbp genome contains 1,604 genes that encode proteins and the remainder include 1,116 pseudogenes as well as non-coding regions [61,62]. The average G+C content was 57.8% [61]. 50% of the genes corresponding to metabolic genes in *M. tuberculosis* have been lost in *M. leprae*, and the *M. tuberculosis* genome is larger than the *M. leprae* genome [63]. In *M. leprae* all major anabolic pathways are relatively intact. However, the genes encoding the lipolysis pathways used to digest host lipids and fatty acids for energy have been extensively downsized [63].

Since the genes that contribute to lipid biosynthesis and metabolism are pseudogenes, *M. leprae* depends on the host cell lipid metabolism to survive. Of the 24 genes encoding Lip lipolytic enzymes in *M. tuberculosis*, only ML0119c (lipE), ML0314c (lipU), and ML1899 (lipG) are conserved in *M. leprae*. Similarly, the central and energy-related metabolic pathways are damaged, and therefore *M. leprae* cannot use common sources of carbon such as galactose and acetate to produce ATP from NADH oxidation. In addition, all biosynthetic and transport systems including microaerophilic and anaerobic electron transfer, as well as the complementary enzyme groups, have been lost in the *M. leprae* genome. This loss implies that the catabolic capacity of *M. leprae* is limited, and the bacterium can only employ a minimal number of carbon sources for growth [62,64,65]. Genes that are necessary for host infection, establishment, and survival are preserved in *M. leprae*, but the genes associated with a parasitic lifecycle dependent on the host have been discarded. In particular, *M*. *leprae* relies on host genes for various metabolic mechanisms, including those involved in lipid metabolism that is essential for cell wall synthesis. This parasitization of host cell genes could explain the extremely slow growth rate of the bacteria and the difficulties with culture using various types of normal media. Meanwhile, *M*. *leprae* is immunologically concealed in host cells, which may facilitate the long-term evasion of host immune surveillance mechanisms.

Many *M. leprae* pseudogenes arose from stop codon insertions that may have been caused by sigma factor dysfunction or the insertion of transposon-derived repetitive sequences [66]. Investigation into *M. leprae* gene structures indicated the existence of multiple stop codon insertions and fragmented ORFs, precluding the translation of these genes into functional proteins [67]. Over 26 extinct insertion sequences (ISs) and four series of distributed repetitive sequences have been confirmed in the *M. leprae* genome, namely *RLEP* (37 copies); *REPLEP* (15 copies); *LEPREP* (8 copies); and *LEPRPT* (5 copies) [68]. The decrease in the genome size of *M. leprae* is largely due to the recombination of these repetitive sequences, which contribute almost 2% of the *M. leprae* TN genome. *RLEP* is often present within pseudogenes and at the 3’-termini of genes, and is often utilized as a PCR target to detect *M. leprae*. These sequences were suggested to be the vestiges of transposons that had lost the ability to go through transposition [69].

There is little detailed information regarding the functional role of pseudogenes and non-coding regions in *M. leprae*. However, some RNAs encoded by these regions are expressed as RNA, and these RNAs can have variable expression levels after infection and among patients with leprosy [70–72]. Among the RNA transcripts, 36% are from pseudogenes, and 43% of all pseudogenes are transcriptionally active [66,67]. In their detailed analysis of RNAs expressed in the *M. leprae* genome, Suzuki et al. revealed that many RNAs are transcribed from pseudogenes and untranslated regions [71]. Using tiling arrays, Akama et al. demonstrated that of the known RNA transcripts, fewer than half (30.1%) arose from coding genes, whereas 37.3% were derived from pseudogenes and 32.5% were derived from non-coding regions [66,73,74]. An essential characteristic of the *M. leprae* pseudogenes is that between one and 40 stop codons are present in-frame. The majority (75%) of pseudogenes that are transcribed do not have a conventional start codon, but 67% contain five or more stop codons [70]. The signal intensity attributed to these non-coding regions was higher than that obtained from the pseudogenes. Repetitive *RLEP* sequences specific to *M. leprae* and sequences with no homology to identified functional non-coding RNAs are included in the non-coding regions [66]. Although the biological significance of these RNAs is not known, they are useful in disease diagnosis and in determining treatment efficacy in assays employing molecular techniques such as PCR in skin-slit smears [72,75,76].

### Genotypes

Genotyping with single nucleotide polymorphisms (SNPs) and short tandem repeats (STRs) is useful to elucidate the transmission and origin of *M. leprae*. Globally, approximately 32 STR loci have been employed for the strain typing of *M. leprae* [77–80]. *M*. *leprae* branches are classified as specific SNP types or subtypes [81], including 4 SNP types (1 to 4) and 16 SNP subtypes (A to P) [82,83], that provide information about the global distribution of leprosy [83,84]. Consequently, many analyses were performed as to the origin and global distribution of leprosy on the basis of variable number tandem repeats (VNTRs) and SNPs [85–91]. Branch 0 is the most ancestral branch and contains bacteria corresponding to the SNP subtype 3K. It is primarily found in Eastern Asia (China, New Caledonia, and Japan). Meanwhile, Branch 1, which is mainly observed in Eastern and Southern Asia, corresponds to the SNP type 1 [83,92–95]. SNP type 2, including branch 2, is mainly found in South Asia and the Near East [83,92,96]. Branch 3, which corresponds to the SNP subtype 3I, occurs in Latin America. Recently, Branch 3 bacteria have been spreading among the nine-banded armadillo in the southwestern United States [97] with zoonotic transmission observed infrequently [85]. Branch 4, which corresponds to the SNP type 4, has been detected in West Africa and South America [83,95,98]. The SNP types 1 and 3 predominate in Thailand and Myanmar but not in Japan, Indonesia, or Korea [99].

The potential for *M. leprae* to have a long incubation period (as long as 30 years) was demonstrated in a study by Suzuki et al. The authors reported a 31-year-old female chimpanzee who was diagnosed with leprosy in Japan. In 1980, this chimpanzee was 2 years old and was trapped in Sierra Leone, West Africa, then sent to Japan. PCR amplification and direct sequencing were performed for SNP analysis to decide the origin of this case. The genotype of the bacilli had only been identified in West Africa, and had not been detected in Japan. It was classified as the SNP type 4 [100,101]. Natural leprosy cases in other nonhuman animals have been identified, but it is not clear whether they were infected by humans or by another nonhuman animal. Sequencing and phylogenetic analyses to compare the entire *M. leprae* genomes which were identified from nonhuman primates and humans from around the world have been carried out. These analyses show that an *M. leprae* strain from a cynomolgus macaque and a human *M. leprae* strain of New Caledonia are closest in lineage. However, the *M. leprae* strains found in chimpanzees and sooty mangabeys are closely affiliated with a West African human *M. leprae* strain [102].

## Immune reactions and animal models of disease

### Innate and acquired immune responses

**Figure 2.**
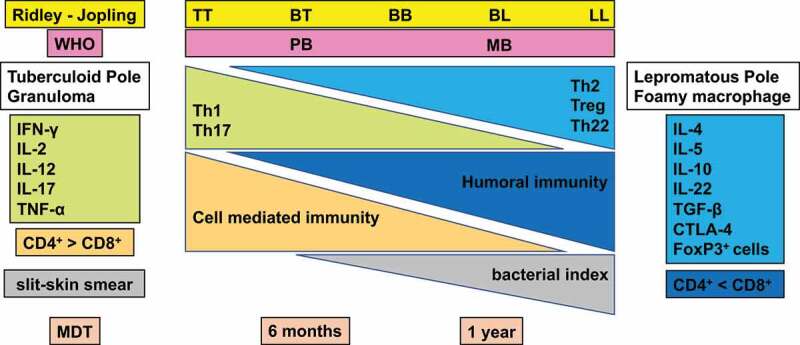
Classification of leprosy on the Ridley-Jopling scale based on immunology, histology, and bacteriology. Leprosy is classified into tuberculoid (TT), borderline tuberculoid (BT), mid-borderline (BB), borderline lepromatous (BL), and lepromatous leprosy (LL). TT leprosy showing strong cell mediated immunity is characterized by granulomatous skin lesions infiltrated predominantly by lymphocytes and epithelioid cells along with high secretion of the Th1 cytokines IL2, and IFN-γ in TT/BT lesions. Conversely, in BL/LL a high level of humoral immunity with a low level of cell mediated immunity is exhibited predominantly by macrophage granulomas with foamy macrophages with few lymphocytes, high levels of Treg cells along with numerous acid-fast bacilli and secretion of high levels of IL-4, IL-5 and IL-10.

The spectrum of clinicopathological manifestations in leprosy arises due to variable host immune responses to *M. leprae* [21,103]. Immune responses include the identification of the pathogen, selection of an adequate immune response, repression of damaging or inadequate immune responses, and over expression of an immune response which induces tissue damage. The host eliminates or controls the pathogen to restore homoeostasis and to prevent tissue damage. While, the pathogen is normally eliminated by an immune response, an inadequate immune response against *M. leprae* causes the parasite to persist without tissue injury of the peripheral nerves and skin.An excessively strong immune response can also cause damage. Thus, host immune systems influence the clinical presentation of leprosy [104,105]. Histologically, granulomatous local skin lesions together with strong cellular immunity and low humoral immunity are characteristic of TT. In contrast, LL is characterized by strong humoral immunity and exhibits tissue reaction by forming macrophage granulomas with only a few lymphocytes [21,106] (Figure 2). However, the elimination of *M. leprae* by humoral immunity is difficult. This contributes to the progression of the disease [107,108].

Initial recognition of *M. leprae* by host cells occurs via pattern recognition receptors (PRRs), including Toll-like receptors (TLRs) [109–111] and NLRs [112–114]. *M. leprae* lipoproteins are ligands of the TLR2/1 heterodimer [115,116] and TLR2/6 heterodimer [117,118]. PGN, LAM and zymosan, found in the cell walls of bacilli, are TLR2 ligands [111,119,120]. *M. leprae* activates TLR2/1 heterodimers of skin macrophages, stimulating the killing of *M. leprae* [120]. *M. leprae* can also activate innate receptors such as TLR4 [121]. However, the outermost cell wall component of *M. leprae*, PGL-I, dampens TLR4 signalling pathway activity in macrophages by downregulating protein expression of the TIR-domain-containing adapter-inducing interferon-β (TRIF), a TLR4 adaptor.

In human alveolar epithelial cells, *M. leprae* also induces IL-8 secretion through the TLR9/NF-κB pathway [122]. IL-8 mediates mobilization of leukocytes to sites of infection in the airway and helps protect the host against invading *M. leprae*. NOD2 is known as an another PRR that participates in the detection of *M. leprae* [113,123]. The human NOD2 receptor induces an innate immune response via IL-32, signalling monocytes to differentiate into dendritic cells [113,114].

The characteristic immune response in patients with TT is the Th1 cytokine response with production of IFN-γ and IL-12, which induces cell-mediated immunity. High *T*-cell responses also lead to *M. leprae* clearance in the skin lesions [124–126]. In TT cases, the activation of macrophages occurs and the formation of epithelioid cells, a cell type in which CD4+ T cells predominate, takes place. *M. leprae*-specific humoral immunity has rarely been detected in TT cases [124,127]. On the other hand, a characteristic of LL cases is the Th2 cytokine response with production of IL-4, IL-5 and IL-13. This signalling increases the humoral immune response to yield strong production of antibodies. But even with massive antibody production and the formation of immune complexes, the growth of *M. leprae* in skin lesions cannot be inhibited [128]. In LL cases, patients have fewer CD4+ T cells compared to those with TT, but the infiltration of many CD8+ T cells is observed. Macrophages which are infected with *M. leprae* as well as foamy macrophages in widespread skin lesions can also be seen [124,127,129–132] (Figure 2).

Only a few studies have investigated T regulatory (Treg) cells in leprosy and there is no consistent theory regarding their roles in leprosy cases. The presence of Tregs in skin lesions in leprosy is known [133]. A relevant role for Tregs in type 1 reactions and their elevation in TT cases were reported [134–136]. Additionally, the number of peripheral blood mononuclear cells (PBMCs) which were stimulated with *M. leprae* cell wall antigen from BL and LL cases was higher than that for borderline tuberculoid BT and TT cases. This result suggests that Treg cells could be associated with the survival of *M. leprae* [136,137]. The expression of IL-10 and cytotoxic T lymphocyte antigen-4 (CTLA-4) and the Treg cell count are higher in LL than in TT cases [136]. On the other hand, a significantly higher frequency of Th17+ cells was noted in BT/TT patients as compared to BL/LL patients. Th17+ cells produce IL17-A, IL17-F, IL-21 and IL-22 leading to tissue inflammation and tissue damage due to neutrophil recruitment, macrophage activation and the activation of Th1 cells [137–140]. These findings suggest that Th17+ cells have a protective function against *M. leprae* infection [124]. However, patients with leprosy reactions showed higher expression levels of cytokines related to Th17 [141]. During leprosy reactions, patients show increased expression and release of the pro-inflammatory cytokines IL-6 and transforming growth factor (TGF)-β that coordinately induce Th17 differentiation. Since Treg and Th17 have opposing functions, it can be speculated that an imbalance in Treg and Th17 differentiation leads to the immunopathology seen with leprosy reactions [137,142].

### Role of macrophages

Macrophages have an important role in leprosy pathogenesis mediate interactions between the host and *M. leprae*. Macrophages are classified as M1 (pro-inflammatory) and M2 (anti-inflammatory) according to the Th1-Th2 dichotomy. These macrophages differ in their cell surface markers, cytokine secretion and biological functions [143,144]. A characteristic of M1 macrophages is their enhancement of antimicrobial, inflammatory and antigen-presenting activities. Additionally, M1 macrophages are activated by proinflammatory cytokines, e.g. IFN-γ. M2 macrophages, which have anti-inflammatory actions, are related to the repair of tissue along with fibrosis, and are activated by IL-4 and IL-13 [144,145]. *M. leprae* is phagocytosed by macrophages, a process that is facilitated by the complement receptors CR1 (CD35), CR3 (CD11b/CD18) and CR4 (CD11c/CD18) [146]. LL skin lesions are characterized by heavily infiltrated foamy macrophages. In this disease type, *M. leprae* multiplies in lipid-filled phagosomes in foamy macrophages. On the other hand, in TT lesions, M1 macrophages activate the classical pathway, leading to the induction of IFN-γ, TNF-α, and iNOS [147]. These cytokines may contribute to immune responses to *M. leprae* [148,149]. A potential regulating factor in leprosy as it pertains to macrophage polarity is the protein jagged 1 (JAG1) [150]. Further, endothelial cells without stimulation lead to M2 macrophage polarization, but endothelial cells activated by IFN-γ give rise to M1 macrophages. JAG1 is present in the vascular endothelium of TT skin lesions. It triggers M1 antimicrobial macrophage differentiation [150]. Therefore, adequate signalling from endothelial cells to monocytes leads to an effective response against *M. leprae* infection. Conversely, a predominance of M2 macrophages promotes anti-inflammatory reactions that can be observed in LL lesions. M2 macrophages induce the production of TGF-β, IL-10, fibroblast growth factor (FGF)-β, CD163, CD209, arginase 1, and indoleamine 2, 3-dioxygenase (IDO), which is involved in immunosuppressive reactions and repair of tissue [151–153]. IL-10-programmed macrophages that play a role in lipid uptake [154] are typified by a strong expression of CD206 (mannose receptor) and scavenger receptors, including CD163 (haemoglobin scavenger receptor), CD204 (scavenger receptor A: SR-A), CD36 and macrophage receptors with a collagenous structure (MARCO) [155]. Uptake of M. *leprae* by CD209+CD163+ macrophages was also demonstrated with LDL in foam cells of LL lesions, suggesting a function for IL-10 derived macrophages in lipid uptake [155].

The association of M4 macrophages with the pathogenesis of atherosclerosis was recently acknowledged and studied in relation to leprosy [156,157]. The immunolabeling of markers of M4 macrophages, including CD68, S100A8 and MMP7 [158], revealed numbers that are higher in LL lesions compared to TT lesions [157]. The phagocytosis of M4 macrophages was suppressed and may be related to low CD163 expression levels [150]. The predominance of M4 macrophages, which are associated with atherosclerosis, may affect foam cell formation, indicating the onset of an oxidative stress reaction. This promotes the production of chemokines and monocyte recruitment. The emergence of Virchow cells is part of an adaptive process.

On the other hand, studies of *M. bovis* Bacille Calmette-Guérin (*M*. *bovis* BCG) infection indicated that the tryptophan aspartate-containing coat protein (TACO; also termed coronin-1 or CORO1A) accumulates on the phagosome membrane and inhibits phagosome-lysosome fusion to enhance intracellular survival [159]. Following phagocytosis, macrophage mycobacteria-containing phagosomes mature with lysosomes to kill engulfed bacteria. However, intracellular *M. leprae* suppresses immune responses and can survive in macrophages. We observed that after *M. leprae* infection, CORO1A was recruited to phagosomal membranes where it suppresses TLR-mediated innate immune activation in cultured macrophages. We also found that the innate immune response, which is activated by TLR2, suppresses CORO1A expression [160,161]. *M. leprae* infection also suppresses NF-κB activation [161]. This escape of various bactericidal actions likely allows the survival of *M. leprae* in host cells for long periods.

### Animal models

There have been numerous studies aiming to grow *M. leprae in vitro* or in animals, and to find an animal model of leprosy to study the pathogenesis and treatment of *M. leprae* infections. Animals used for this purpose include various birds, mammals, and cold-blooded animals [162]. Most attempts resulted in no host response or only mild inflammation at the inoculation site. This outcome may have been due to a lack of an innate response by the host to *M. leprae*. The high body temperature (≥37°C) in traditional rodent models may also disturb *M. leprae* proliferation, thereby making it difficult to establish conventional animal models to study *M. leprae* pathogenesis [163]. An initial success of transmission for limited infection in animals was achieved in 1960 by Shepard et al. in mouse footpads [164], and was based on the preference of *M. leprae* for cooler areas of the body. Although immunocompetent mice are quite resistant to *M. leprae* infection, inoculation with 10^3^-10^4^ bacilli into the posterior footpad of BALB/c, B6, or CFW mice resulted in local growth with a plateau at about 10^5^-10^6^ organisms within 4–6 months [165]. However, with the inoculation of 10^6^ bacilli into each mouse footpad, the number of bacteria did not increase. This result suggests that proliferation could occur when the inoculum contains few bacilli. Thus, inhibition of bacterial growth was not because of the low temperature of the inoculation site, but because of cellular immunity [166,167]. The inoculation of immunocompromised (thymectomized-irradiated (*T*-R)) mice resulted in 10- to 100-fold more growth than that seen in immunocompetent mice.

In the first few months post-infection, host reactions against *M. leprae* are assumed to be largely under the control of innate immunity. At the peak or plateau stage of growth, the onset of adaptive cellular immunity takes place, which results in the death of the bacilli. In immunocompetent mice, a moderate granulomatous reaction with a mild lymphocytic infiltrate and a small number of epithelioid cells and histiocytes resembling a TT lesion can be seen. In contrast, *M. leprae* spontaneously grows in the footpads of athymic nude mice, which lack cell-mediated immune responses, and granuloma formation similar to that seen for LL occurs in athymic nude mice [168]. In infected footpad tissue, giant lepromas and many bacilli can be seen in the histiocytes in the absence of mature T cells. Other models such as severe combined immunodeficiency (SCID) mice and mice having gene-knockouts (KO) of IFN-γ and nitric oxide synthase 2 (NOS2) [168] failed to reproduce human leprosy-like regions [169–171]. Meanwhile, a congenic hypertensive nude rat SHR.F344-Foxn1^*rnu*^ carrying nude (*rnu*) and hypertension genes had high IL-10 production and high susceptibility to *M. leprae* [172,173]. Inoculation of these nude rats with *M. leprae* induces leproma patterns in the inoculated and non-inoculated sites. Although this model is not widely used, it may be useful as an animal model for leprosy, particularly LL leprosy. Successful *M. leprae* growth in this model suggests a possible link between *M. leprae* growth and a genetic background of hypertension or other abnormalities of systemic metabolism.

The nine-banded armadillo (*Dasypus novemcinctus*) is a natural host and reservoir of *M. leprae* in the United States [85,99,174]. In 1971, the first successful inoculation of *M. leprae* in the nine-banded armadillo was reported [175]. This animal model has extensive neurological involvement with *M. leprae*. Thus, the armadillo is useful to understand mechanisms of neuropathy and to investigate new therapeutic interventions [176,177]. A limitation is that armadillos do not breed in a laboratory environment and must be captured from the wild [178]. The zebrafish (*Danio rerio*) is a useful model for the study of *M. tuberculosis* granulomas using *Mycobacterium marinum* (*M. marinum*) [179]. Recently, *M. leprae*-induced granulomas and early nerve damage were studied in adult zebrafish [180]. These animal models, although limited, have allowed for basic research on the pathogenesis of *M. leprae* and the epidemiology and therapeutic approach to leprosy [96,181–183]. Among primates, procedures for intravenous and intradermal inoculation with *M. leprae* were established in more than half of sooty mangabey monkeys (*Cercocebus atys*) and African green monkeys (*Chlorocebus aethiops*) studied, but experiments with rhesus monkeys (*Macaca mulatta*) and Cynomolgus monkeys (*Macaca fascicularis*) were less successful [184,185].

## Host interactions

### Route of transmission

*M. leprae’s* transmission routes are not completely known, although an increased risk of human-to-human transmission because of close contact with untreated leprosy patients has been noted. The most likely candidate for transmission is the spread through infectious aerosols [186]. Although several reports described the detection of *M. leprae* in placental tissue, congenital transmission of leprosy has not been established [186,187]. Most reported cases of leprosy in very young infants can be explained by exogenous transmission through airborne infection of newborns from mothers or other untreated leprosy patients. The term of incubation is known to be long, as is the time for disease onset [100,188,189]. In the southeastern part of the United States, there have been reports of zoonotic transmission from wild infected armadillos, which seems to be the cause of autochthonous transmission in this area [85,97,190]. Some cases of transmission of leprosy through armadillos were reported in Latin America including Venezuela, Colombia, Brazil and Mexico, although the transmission between armadillos and humans is very rare [191]. Recently, British red squirrels (*Sciurus vulgaris*) were found to harbor *M. leprae* and *M. lepromatosis* [192,193].

*M. leprae* has also been detected in wild chimpanzees (*Pan troglodytes*) and sooty mangabey monkeys [100–184–194–198]. Leprosy-like symptoms, such as loss of hair and hypopigmentation of facial skin, along with nodules on the face and other areas, have been observed. Deformations and ulcers of the hands (claw hand) and of the feet were reported in two groups of wild Western chimpanzees native to a national park in West Africa [198]. Fecal and necropsy samples of these animals showed that *M. leprae* is the causative pathogen. Phylogenomic analysis and comparison to strains from other animals, including humans, allowed the identification of the rare bacterial genotypes 4N/O and 2F among the chimpanzees. There have not been any cases of genotype 2F in West Africa and very few cases in Ethiopia. In West Africa, there was only one case of genotype 4N/O that was found in a human sample. Despite the prime hypothesis that *M. leprae* infection could occur through chimpanzee-to-human transmission, this phylogenomic analysis suggested a possible distribution of *M. leprae* in the wild environment. Recently, potential reservoirs have been identified. It is possible that *M. leprae* could be delivered through tick bites [199–201], and *M. leprae* can survive in amoebae and in kissing bugs (*Rhodnius prolixus*) [202]. Further research is required to establish the role of these vectors in *M. leprae* transmission [203,204].

**Figure 3.**
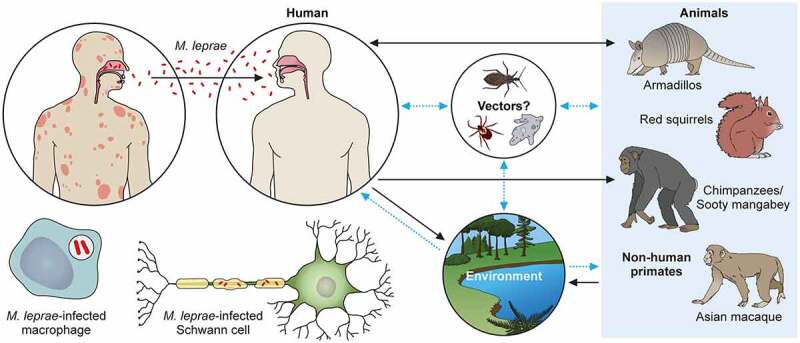
The transmission pathways of *M. leprae*. The *M. leprae* transmission pathways are not fully clear. However, an increased risk of human-to-human transmission because of intimate communication between untreated leprosy patients has been noted. Spreading *via* infectious aerosols is considered to be the most likely route of infection. *M. leprae* invades skin macrophages and Schwann cells, inducing skin lesions and neurological injury. Zoonotic transmission of *M. leprae* due to natural infection of armadillos in the Southeast United States has been reported, and humans and armadillos share a specific *M. leprae* strain. Red squirrels (*Sciurus vulgaris*) in the British Isles harbour *M. leprae*. Non-human primates including chimpanzees (*Pan troglodytes*) have been detected with leprosy in Africa and Asia. It has been speculated that potential vectors, such as amoebae, kissing bugs, and ticks, as well as the environment, could be potential transmission routes for *M. leprae* as a zoonotic disease. Black dotted arrows show confirmed transmission pathways. Grey arrows show hypothetical transmission pathways. Red dotted arrows show the main route of transmission between humans. An aerosol spreads the nasal secretions.

Regarding the relationship between the environment and infection, the detection of mRNA indicated the existence of the bacilli in soil and water samples taken in India and Brazil [205,206]. The results showed that the SNP subtype (SNP type 1and subtype 1-D from India and subtype 4-N from Brazil) of *M. leprae* from skin biopsies coincided with those identified in environmental samples. The confirmed and hypothetical transmission pathways are shown in Figure 3 (same as graphical abstract).

### Role of Schwann cells in nerve injury

*M. leprae* infects from the outside of nerves likely through epineural blood vessels and lymphatics [207,208]. *M. leprae* can reach the endoneurium via blood vessels and then invade and proliferate within Schwann cells [177]. *M. leprae* invades Schwann cells through α-dystroglycan (DG) which is present in the G domain of the laminin (LN)-2 α2 chain of the basal lamina [209,210]. Schwann cells and *M. leprae* interact through the surface moieties of the bacteria, including PGL-I, which has an important role in adhesion and survival within Schwann cells. Histone-like protein (Hlp) [211] and LPS-binding protein (LBP-21) [212] also participate in the interaction.

*M. leprae* induces neural demyelination by stimulating the production of matrix metalloproteinases (MMPs), C-X-C motif chemokine ligand 10 (CXCL10) and C-C motif chemokine 2 (CCL2), which attract macrophages. PGL-I induces nitric oxide (NO) synthase production in infected macrophages that results in damage to axons caused by mitochondrial injury and the induction of demyelination [180,213]. Nitrotyrosine, which is the metabolism product of NO, is seen in the nerves of BL patients. Nitrotyrosine is also involved in the lipid peroxidation of myelin, which leads to the demyelination of nerves [214]. Also, it has been reported that the direct mechanism of *M. leprae*-induced demyelination depends on pathogen binding to the receptor-type tyrosine kinase ErbB2 on Schwann cells and the resulting activation of Erk1/2 signal transduction pathways through the Ras-Raf-MEK-ERK pathway [215,216]. T cells are known to kill Schwann cells by cell-mediated immunity [131,217] and other cytokines, like IL-1β, IFN-γ, and TNF-α lead to apoptosis in cultured Schwann cells [218,219]. In addition, the complement system is involved in the demyelination seen in leprosy patients, which is termed rapid Wallerian degeneration. Recent findings showed that nerve damage observed in the early phase may be associated with the innate immune response. The complement membrane attack complex (MAC), which consists of C5b, C6, C7, C8 and C9, colocalizes with LAM of the *M. leprae* cell wall in the nerves of patients with leprosy, indicating that complement targets LAM [220]. MAC deposition occurs on the sensory nerves of the skin and on damaged sensory nerves of LL patients, but not of TT patients [220,221].

*M*. *leprae* induces significant changes of the metabolic pathways in Schwann cells, such as inducing insulin-like growth factor 1 (IGF-1) which enables bacteria to survive in host cells [222]. In addition, *M. leprae* upregulates glucose uptake and lipid synthesis while downregulating oxidative stress, apoptosis, and autophagy [223–225]. In host cells, PGL-I activates endocytic mannose receptor (MR/CD206) expression depending on the peroxisome proliferator-activated receptor gamma (PPAR-γ). Crosstalk between CD206 and PPAR-γ leads to the formation of lipid droplets (LDs) and produces prostaglandin E2 (PGE2) [226]. Meanwhile, TLR6 has a role in bacterial sensing and entry in Schwann cells [224]. It has been shown that *M. leprae* Man LAM is recognized by CD206, resulting in bacterial invasion, weak activation of PPAR-γ, and upregulation of CD206 expression. *M*. *leprae* amplifies CD206/PPAR-γ crosstalk to promote LD formation and phagosome recruitment. Elevated LDs induce PGE2 and IL-10 production [224].

*M.leprae* induces the Schwann cell dedifferentiation by promoting early demyelination. The bacteria may further promote the spread of infection by reprogramming Schwann cells to the progenitor/stem cell stage [227]. The infection with *M. leprae* stimulates the expression of multiple immune-related genes related to innate immunity [228]. Schwann cells which are infected with *M. leprae* for an extended period showed early demyelination followed by increasing Schwann cell number. Further dedifferentiation results in the loss of cell lineage characteristics and the formation of progenitor/stem cell-like cells (pSLCs) having an expression profile like that of mesenchymal stem cells. These pSLCs can differentiate into fibroblasts and can undergo reprogramming that allows spread of the infection [227,228].

### Lipid accumulation in infected cells

Mycobacteria cell walls contain a large range of lipids. As stated above, *M. leprae* has lost approximately 50% of the lipid metabolism-related genes of *M. tuberculosis* [64,229]. Thus, *M. leprae* may have also lost the ability to metabolize lipids needed to maintain complex cell walls and as a carbon source. Microscopic analysis of skin biopsies from LL cases showed that phagosomes containing *M. leprae* co-localized with LDs in foam cells [230]. Recently, we used high-performance thin-layer chromatography (HPTLC) to indicate that triacylglycerol (TAG) is the major lipid of human monocyte THP-1 cells infected with *M. leprae* [15]. The accumulation of TAG was sustained with live bacilli, but was temporary when dead bacilli were used. *M. leprae* promotes glucose uptake by increasing the expression of the solute carrier family 2 member 1 (SLC2A1). SLC2A1 activates the pentose phosphate pathway and provides TAG synthesis in Schwann cells [231]. TAG is associated with adipose differentiation-related protein (ADRP) and perilipin, which regulate lipid metabolism [232]. We previously showed that there is upregulation of ADRP and perilipin in foamy macrophages involving *M. leprae* in skin lesions [111]. The expression of perilipin and ADRP is highly promoted by *M. leprae* infection, which is in agreement with *in vivo* data. These proteins are found in *M. leprae*-containing phagosomes in THP-1 cells [111]. On the other hand, *M. leprae* suppresses TAG degradation by inhibiting the revelation of hormone-sensitive lipase (HSL), therefore promoting an intra-host macrophage environment that contains high levels of lipids [72]. TAG biosynthesis occurs mainly through a *de novo* pathway that involves the glycerol-3-phosphate pathway [233]. In that pathway, glycerol-3-phosphate acyltransferase (GPAT) functions as a rate-limiting enzyme of TAG biosynthesis. *M. leprae* infection causes the synthesis of TAG within host cells by inducing expression of GPAT3 among four isoforms (GPAT1–4) [15]. This finding is supported by the observation that THP-1 cells with CRISPR-Cas9-mediated GPAT3 KO had dramatically reduced TAG accumulation after *M. leprae* infection. These observations indicate that *M. leprae* changes lipid metabolism of the host cell to sustain TAG accumulation. *M. leprae*-containing histiocytes and Schwann cells were also reported to be filled with cholesterol [225,234], which was confirmed by TLC showing that cholesterol accumulates in *M. leprae*-infected primary macrophages [225,230]. In addition, the expression of 3-hydroxy-3-methylglutaryl-CoA reductase (HMGR) increased in host cells, and lovastatin inhibition of HMG-CoA reductase reduced the *M. leprae* survival rate [230]. Thus, the viability of *M. leprae* is likely to be associated with lipid metabolism in host cells, especially TAG and cholesterol storage.

After infection, *M. leprae* causes dramatic changes in host gene expression profiles [72,76,111,231,235]. PPARs, which include PPAR-α, PPAR-β/δ, and PPAR-γ, which are major regulators of lipid metabolism, have important roles in the formation of LDs containing TAG. We showed that PPAR-δ and PPAR-γ expression is induced by *M. leprae* infection and nuclear translocation in THP-1 cells [76]. Activation of these genes by *M. leprae* induced the expression of genes encoding CD36 and the acyl-CoA synthetase long-chain family (ACSL). CD36 mediates the macrophage uptake of oxidized low-density lipoproteins (LDL) and induces the expression of fatty acid-binding protein 4 (FABP4) which promotes foam-cell formation through fatty acid transport. ACSL is associated with *de novo* TAG synthesis from intracellular fatty acids [76,235]. Also, PPAR-β/δ and PPAR-γ regulate GPAT3 and ADRP expression [236,237], but the components derived from viable bacteria that act as triggers of these PPARs are unknown. Since lipids accumulated in host cells are essential for *M. leprae* survival, drugs that target lipid metabolism could be a new therapeutic strategy for leprosy.

As mentioned above, mycolic acid is a cell wall lipid that is unique to mycobacteria and is a key virulence factor [238]. Mycolic acid primarily exists in combination with trehalose, glucose, and glycerol. Subtypes of mycolic acid in *M. tuberculosis* include alpha-, keto-, and methoxy-mycolic acid based on functional groups within the mero mycolate chain. Meanwhile, *M. leprae* has alpha- and keto-mycolic acids [239]. The structure of these mycolic acids contributes important characteristics to the different bacteria including resistance to chemical damage and dehydration, low permeability to hydrophobic antibiotic substances, and the ability to survive within phagosomes [240]. *Mycobacterium smegmatis* (*M. smegmatis*) synthesizes mycolic acid using an extension reaction from acyl-CoA *via* fatty acid synthase I and II (FASI and FASII) as well as a pathway that uses TAG-metabolized fatty acids [241]. Interestingly, inhibition of TAG degradation by anhydrotetracycline (ATc) dramatically reduced mycolic acid biosynthesis, suggesting that *M. smegmatis* stores lipids that could serve as an intracellular fatty acid reservoir for biosynthesis of complex lipids [241]. As we have reported, TAG accumulated in *M. leprae*-infected macrophages contains a complex mixture of fatty acids and may produce materials for the synthesis of mycolic acid. Further studies are needed to investigate whether *M. leprae* synthesizes mycolic acid from host-derived TAGs to escape host-derived exclusion systems and allow intracellular parasitization.

## Clinical characteristics and epidemiology

### New cases

According to the WHO, there were 202,185 new leprosy cases worldwide in 2019. India had the highest number, followed by Brazil and Indonesia. Of these, 14,981 cases were in children younger than 15 years old. G2D, which is defined as leprosy accompanied by visible deformities due to leprosy neuropathy, was detected in 10,813 cases. The COVID-19 pandemic hampered implementation of the WHO program and there were 37% fewer new cases reported in 2020 than in 2019 [242], although whether there was an actual decrease in incidence is unclear.

### Classification

Leprosy can be classified into five types: TT, BT, BB, BL and LL by the Ridley-Jopling scale. This classification is based on clinical, immunological and histological differences in the disease [128]. Dimorphic cases are classified according to which pole they move towards (TT or LL) and are preceded by the word borderline (BL, BT and BB) [29] (Figure 2). Most of the indeterminate cases are known to be cured without treatment. Dimorphic cases will upgrade or downgrade on the immunological scale according to the immune status of the host. As treatment is initiated such cases tend to upgrade on the Ridley-Jopling scale [243,244]. The skin smear is used to estimate the number of acid-fast bacteria present. This value is reported as the Bacterial Index (BI) [245]. The WHO Expert Committee on Leprosy established a practical classification system that can be applied worldwide in 1998 [6]. Cases having six or more skin lesions are classified as multibacillary (MB), and those with five or fewer skin lesions are classified as paucibacillary (PB). The classification of PB and MB was adopted for easy administration of MDT under field conditions. The skin manifestations and nerve damage caused by leprosy are strongly influenced by the immune response of each patient and may show a variety of clinical manifestations and histopathologic morphology [128,246,247].

### Histopathology

**Figure 4.**
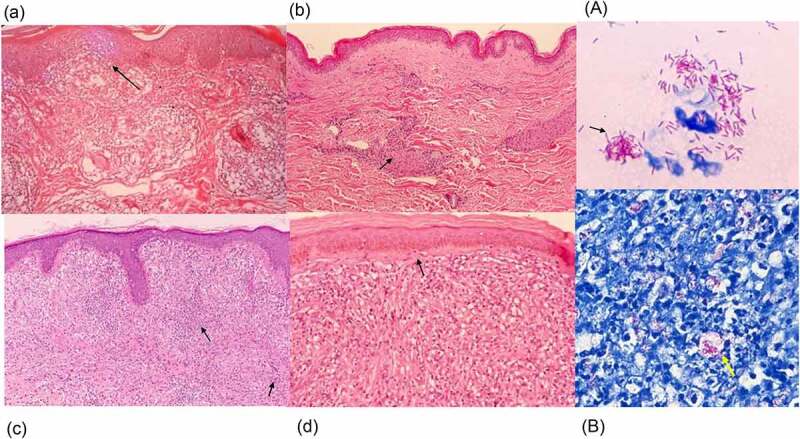
Histopathology of leprosy. (a) TT is characterized by granulomas with lymphocyte infiltration. These are multiple, well-formed granulomas with multinuclear Langhans giant cells. Erosion of the basal layer of epidermis is observed, with lymphocytes (→). (HE stain, 40×). (b) in BT lesions a granulomatous appearance can be observed (→), similar to TT lesions, with the presence of a grenz zone. Lymphocytic infiltration is less than in TT. (HE stain, 40×). (c) in BL cases, lymphocytic infiltration and histiocytes (→) with granular to foamy cytoplasm are observed. (HE stain, 40×). (d) LL is characterized by foamy histiocytes with a grenz zone below the epidermis. (→) (HE stain, 40×) (A) the slit skin smear test shows the acid fast bacilli. (→) (Ziehl- Neelsen stain, 1000×). (B) a large number of bacilli are observed within foamy histiocytes with LL lesions. (→) (Ziehl- Neelsen stain, Wade-Fite, 400×). Photomicrographs are courtesy of Dr. Norihisa Ishii, National Sanatorium Tama Zenshoen.

On a histological basis, TT is characterized by granulomas with lymphocyte infiltration. These granulomas are composed of epithelioid histiocytes that build multinuclear Langhans giant cells. Erosion of the basal layer of epidermis with lymphocytes can be observed without an intervening clear zone (Figure 4a). Nerve bundles are difficult to identify within the granulomas and S100 immunostaining may be required. In TT lesions, bacilli are not seen. In BT lesions, a granulomatous appearance can be observed with a grenz zone, which is a very narrow space that separates the superficial inflammatory infiltrate and the epidermis; this appearance is similar to that of TT lesions. Lymphocytic infiltration around granulomas is less than that seen for TT and the swelling of nerve bundles may be observed in the granulomas (Figure 4b). In BB cases, diffuse epithelial histiocytes that form small lesions are observed, but diffuse lymphocytic infiltration is present and there are no Langhans giant cells. BI is frequently 3+ to 4+ in BB lesions. In BL cases, lymphocytic infiltration and the presence of histiocytes with a granular to foamy cytoplasm can be observed (Figure 4c) and BI is usually 5 + .In LL cases, foamy histiocytes (Virchow cells) including numerous bacilli (Figure 4b), which in some cases exist in large clumps termed globi, are present, as is a grenz zone below the epidermis. Lymphocytic infiltration is not evident, and nerve bundle damage is often seen. Below the epidermis, often in the grenz zone, is a very narrow space of collagen that separates granulomas and the epidermis [248,249] (Figure 4d). BI in LL is generally on the higher side, often between 5+ and 6 + .In type 1 leprosy reaction lesions, histopathology findings are characterized by oedema with lymphocytic infiltrates and giant cells [250]. In type 2 leprosy reaction lesions, neutrophil infiltration of the perivascular areas in the dermis and subcutaneous tissue is observed. Necrotizing vasculitis may be seen, along with lobular panniculitis [250]. (See section 4. 6 Leprosy reaction)

### Diagnosis

Recently, the diagnosis of leprosy by polymerase chain reaction (PCR) has become a useful and recommended technique. PCR began to be used for the amplification of *M. leprae* DNA about 20 years ago, and field-based assays are now being developed. This technology has come to be used not only for diagnosis, but also for detection of resistant bacteria, identification of the route of infection, evaluation of therapeutic effects, and confirmation of household contacts. It has been verified and reported that multiplex real-time quantitative PCR (qPCR) using two or three genes, namely *RLEP*, 16S rRNA and superoxide dismutase (*sodA*), is effective [251–253].

The antibodies to PGL-I of *M. leprae* can be detected by enzyme-linked immunosorbent assays (ELISAs) and related immunoassays; these have also been used in epidemiological research [254,255]. Immune biomarkers of infection are also of interest for the IgM response to certain antibodies, particularly PGL-I, which is detected in patients’ serum [256,257]. Elevated levels of IgM, IgG1, and C3d were detected in MB patients who developed erythema nodosum leprosum (ENL); these serum markers may be applicable to estimate the risk of developing a reaction [258]. It was also observed that IgG and IgM antibody levels decreased in LL cases in the first year of their treatment [108,259]. Immunoblot and ELISA-based studies of *M. leprae* antigens are useful for diagnosis and evaluation of the effectiveness of antibiotic treatment. Other molecular detection methods for *M. leprae* include analysis of urine for DNA and protein [260] and detection of antigens and antibodies in cerebrospinal fluid (CSF) [261,262]. In current clinical practice, the diagnosis of leprosy is based on the presence of at least one of three cardinal signs: (i) the loss of sensation in a reddish or pale (hypopigmented) area of skin; (ii) a thickened peripheral nerve with weakness and/or loss of sensation of the muscles; or (iii) the detection of acid-fast bacilli in a slit-skin smear [263,264].

### Neuropathy

Neuropathy is an important clinical manifestation of leprosy and has a large impact on affected individuals. Nerve thickness is a prominent clinical manifestation of leprosy. In the WHO classification [6], PB is defined as having no peripheral nerve damage or is limited to a single nerve, whereas MB has one or more nerve infiltrates. The characteristic of pure neuritic leprosy (PNL) is the damage of peripheral nerve trunks without skin symptoms. In PNL, the ulnar nerve is most frequently influenced, with enlargement of the nerve accompanied by loss of function [265]. In India, 4–18% of leprosy cases are PNL, and in Brazil PNL accounts for 7.8% of cases [266–269]. Mononeuritis with sensory neuropathy of the upper extremities is more frequent than the lower extremities. PNL can be difficult to diagnose because there are no skin lesions, and diagnoses are often delayed. 60% of patients with LL have nerve damage at the time of diagnosis. Furthermore, nerve damage may occur in 30% cases during the treatment of LL and in 10% of cases, new nerve damage associated with immune reactions occurs after drug treatment [270].

### Leprosy reaction

**Figure 5.**
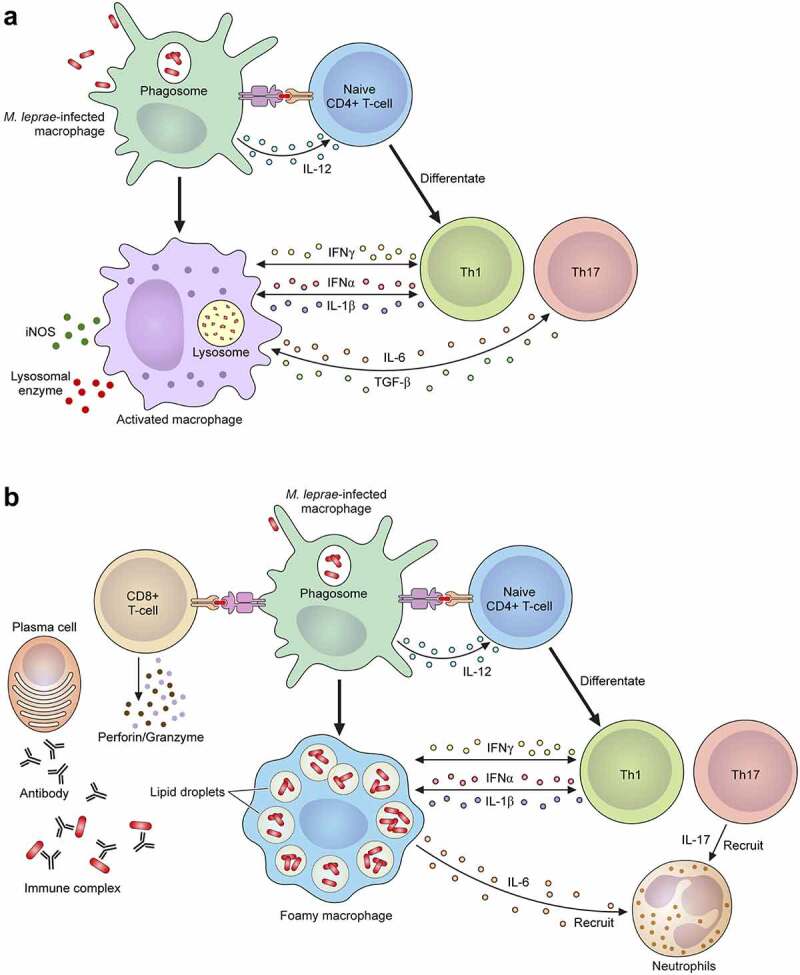
The mechanism of the leprosy reaction. (a) T1 R is led by a cellular immune response mediated via CD4+ T cells. Activated macrophages release pro-inflammatory Th1 cytokines, such as IFN-γ, TNF-α, IL-1β, IL-6, IL-2, IL-12, TGF-β and iNOS, which cause tissue damage. (b) ENL is a generalized proinflammatory reaction featuring the infiltration of neutrophils. The activation of complement, immune complexes, increasing CD4+/CD8+ T cell subset ratios and high degree of proinflammatory cytokines including TNF-α in the lesions and in the circulation can also be observed. ENL shows low cellular immunity, but there are enough B cells and plasma cells to produce antibodies against *M. leprae*. In the acute stage of ENL lesions, a large number of neutrophilic infiltrations is observed. An activated CD8+ T cell secretes cytotoxic granule proteins such as perforin and granzymes which lead to apoptosis of the cells.

Leprosy reaction is the immune response against *M. leprae* and is a major factor in neurological disorders associated with leprosy. This reaction occurs with a sudden and severe immune response to the degraded leprosy bacillus components, which often appear with antibiotic therapy. Leprosy reactions are classified as type 1 (T1 R) and type 2 (also known as erythema nodosum leprosum or ENL). These reactions occur in 30 to 50% of all leprosy patients [271,272]. T1 R is also known as a borderline reaction or a reversal reaction that happens in borderline cases (BT, BB and BL) and some LL cases. Acute inflammation of pre-existing skin lesions with neuroinflammation is a hallmark of T1 R. Approximately 95% of T1 R cases have already started at the time of diagnosis of leprosy or begin in the first two years of MDT [273]. Neurologic dysfunction is present in approximately 10% of PB patients and 40% of MB patients, and is particularly prominent in patients with T1 R [274]. The immune responses related to the pathogenesis of T1 R are a type-IV hypersensitivity reaction [275] and CD4+ T cell infiltration might be responsible for the immune-mediated tissue damage that occurs during T1 R [276]. The Th1 cell-derived cytokines, including TNF-α, IL-2, IL-1β, and IFN-γ, have important functions in T1 R (Figure 5a).

ENL occurs with neutrophil infiltration and systemic inflammation. Complement activation, immune complex formation, increasing CD4+/CD8+ T cell subset ratios and elevated TNF-α may be seen in the lesions and in the circulation [277–279] (Figure 5b). In the acute stage of ENL lesions, large numbers of neutrophilic infiltrations are observed, but the actual function of neutrophils in ENL is unclear. Recent studies showed that neutrophils expressing CD64 are associated with the systemic inflammation of ENL [280], and therefore the CD64 expression level on the neutrophils of peripheral blood may be a marker for ENL and disease severity. ENL occurs in patients with LL or BL who exhibit low cellular immunity but have sufficient numbers of B cells and plasma cells to produce antibodies against *M. leprae* [281,282]. The antibodies form immune complexes that deposit in tissues and blood vessels to cause a type III hypersensitivity reaction [283]. ENL occurs in more than half of LL cases and 5–10% of BL cases, especially where there is a BI value ≥4 [284]. These immune responses are related to TNF-α produced against *M. leprae* itself or to the components of dead bacilli after antimicrobial treatment [284]. In fact, most ENL occur during the first year of MDT [285]. The main ENL symptom is painful red skin lesions that are often soft and subcutaneous. In addition, systemic inflammation which affects the nerves, joints, eyes, testes, and lymph nodes may occur with high fever. Leprosy reactions are the most common source of persistent nerve damage, deformity, and induced disability following *M. leprae* infection.

### Treatment and drug resistance

The current WHO-recommended MDT strategy for leprosy control is a three-drug combination of rifampicin, dapsone and clofazimine given over a 6-month period for PB leprosy and over a 12-month period for MB leprosy. The standard regimen for adult MB patients is a 12-month course consisting of a once-monthly 600 mg dose of rifampicin, 100 mg dapsone daily, 50 mg clofazimine daily and one 300 mg dose monthly [263]. Adult PB patients require a regimen consisting of a 6-month course of 600 mg rifampicin once monthly and 100 mg dapsone daily. For paediatric cases, the dose is based on weight and age, so for a child younger than 10 years old who weighs less than 40 kg, the regimen is: dapsone 2 mg/kg/day, rifampicin 10 mg/kg/day, and clofazimine 100 mg once a month and 50 mg twice weekly. Delays in the diagnosis and required treatment of leprosy often result in deformity and disability, which may result in stigma, and also correlate with the development of G2D [286]. The motor neuropathy that accompanies peripheral neuropathy can cause major problems with ADL and muscle atrophy.

Leprosy treatment with antimicrobials began in the early 1940s, by Dr. Guy Henry Faget of the National Hansen’s Disease Center in Carville, Louisiana. Sulfone therapy (Promin) was replaced with dapsone to limit toxicity of the treatment [287,288]. Dapsone was used as a single agent on a long-term basis until the development of drug resistance was confirmed. The first dapsone-resistant case was reported in 1953 and cases have since been reported [289,290]. In the 1970s, relapses and drug resistance to dapsone occurred in nearly 19% of patients [291]. To avoid the emergence of drug resistance, the WHO recommends MDT as described above, but nevertheless, reports of drug resistance still occur.

Rifampicin is an antibiotic that inhibits transcription in bacteria, especially mycobacteria, by impeding the enzymatic activity of the beta-subunit of RNA polymerase (rpoB) via steric hindrance of the 5’-ribonucleotide of the elongating RNA [292,293]. Missense mutations in the *rpoB* gene lead to changes in the amino acids lining the rifampin binding pocket and produce changes in the enzyme’s structure and rifampin resistance [292]. Within the drug resistance determining region (DRDR), mutations in the *rpoB* gene occur between the codon positions 410–480 [290].

Dapsone is a sulpha drug that does not have a sulphonamide structure but is instead a *p*-aminobenzoate (PABA) analog that competes with PABA as a substrate for dihydropteroate synthase (DHPS). It acts to bacteriostatically inhibit nucleic acid (both RNA and DNA) synthesis in pathogens [294]. The mechanism of *M. leprae* dapsone resistance incorporates mutations found in the *folP* gene encoding DHPS, causing a reduction in binding affinity between dapsone and the DHPS active site [295]. In the mouse footpad assay, clinical isolates from patients who were resistant to dapsone therapy contained missense mutations at codons 53 and 55 in the *folP1* gene [296–298]. Clofazimine is a therapeutic agent used as a first line MDT; however, its mechanism of effect is not fully clear. Over the years, the number of reported clofazimine-resistant leprosy cases has been small. Clofazimine may have several different effects, with high drug accumulation in mononuclear phagocytes, slow metabolic clearance, and anti-inflammatory effects that together may explain why there are few resistant strains [299–301]. Ofloxacin is a fluoroquinolone antimicrobial agent used as part of a second line MDT against *M. leprae*. Fluoroquinolones target type II DNA topoisomerases, such as DNA gyrase and topoisomerase IV [302]. Fluoroquinolone resistance, including resistance to ofloxacin, is conferred primarily by amino acid substitutions in the DRDR near the *N*-terminus of the DNA gyrase A (Gyr A) due to mutations in the *gyrA* gene [303]. Two amino acid substitutions have been reported for ofloxacin resistance, a glycine to cysteine substitution at position 89 of GyrA (Gly89Cys) and an alanine to valine substitution at position 91 (Ala91Val). However, most quinolone-resistant *M. leprae* are Ala91Val substituted strains, with limited resistance to Gly89Cys substitutions [297].

Since the 1970s, rifampicin has been a mainstay of leprosy treatment; however, since the reporting in 1976 of the first case of resistance [304], resistant strains have since been reported in several other endemic areas [305–308]. In the WHO Global Leprosy Programme (GLP), *M. leprae* drug resistance, in particular to rifampicin, was an issue of deep concern. In 2008, in 18 sentinel countries, global-level drug resistance surveillance was performed to evaluate drug resistance levels and MDT efficacy. This surveillance showed that resistant strains are present in many endemic regions across the world [309]. Drug-resistant strains were determined by antibiotic susceptibility testing in mouse footpads, necessitating a one-year term for experimentation. Later, gene sequencing of DRDRs in *M. leprae rpoB, folP1*, *and gyrA* were used to gauge resistance to rifampicin, dapsone, and quinolones, respectively [310–312]. Although the WHO carried out sentinel surveillance using PCR direct sequencing [313], such sequencing analyses or even PCR detection of *M. leprae* DNA in most endemic countries remains challenging. In this regard, development of a rapid, cost-effective point-of-care diagnostic tool is needed.

### Prevention and infection control

The WHO Guidelines Development Group (GDG) analysed evidence regarding vaccines for leprosy [314–316]. Current strategies for vaccine development revolve around using cross-sensitizing mycobacteria. The efficacy of *Mycobacterium bovis* (*M. bovis*) and Bacille Calmette-Guérin (BCG) has been reported in different countries [314]. Three meta-analyses summarized the protective efficacy of BCG vaccination [315,317,318]. It was reported that on average the preventive effect of BCG was 26% in experimental studies, while in observational studies, it was found to be 61% [315]. Recently, India has focused interest on another vaccine, the *Mycobacterium indicus pranii* vaccine, which is known as *Mycobacterium w* (*Mw*) [319,320]. Various recombinant BCGs and *M. leprae* subunit vaccines have been developed, of which the new subunit recombinant vaccine LepVax has successfully completed Phase 1a clinical trials [321].

Allocation of post-exposure prophylaxis (PEP), consisting of a single dose of rifampicin (SDR), is one of the most promising options for people exposed to *M. leprae*. PEP reduces the risk of developing leprosy by about 60% during the first two years after exposure [322–326]. *M. leprae* detected in the nasal cavities of asymptomatic people is a risk for the onset of illness in a community over time [327,328]. The transmission among subclinical patients seems to be suggested by the association of positive asymptomatic residents with infected residents [186]. Individuals in contact with those who are afflicted with leprosy are at increased risk of developing the disease [329,330]. Thus, administration of SDR-PEP to individuals in contact with leprosy patients may help control transmission, thereby reducing new leprosy case numbers [331].

## Conclusion

The Global Leprosy Strategy 2021–2030 “Towards zero leprosy” has been carried out with the goal of targeted reduction of new patients, but a substantial number of infections still occur. This is due to inadequate knowledge about the mechanisms of *M. leprae* pathogenicity and difficulties accessing medical treatment that persist in endemic regions, especially those in Southeast Asia, East Africa, and Brazil, which still account for a large proportion of newly reported cases. This review summarized the unique biological properties of *M. leprae* with a focus on survival within host cells such as macrophages and Schwann cells through modulation of the innate immune response and lipid metabolism. *M. leprae* has extremely few gene-coding regions, and pseudogenes and non-coding regions consume nearly half its genome. Thus, these bacteria are highly dependent on host cells, particularly for the production of lipids and cell wall components. Alterations in lipid metabolism affect bacterial survival and proliferation. Although WHO-recommended MDT administration has drastically brought down the prevalence of leprosy in the world, the emergence of new cases along with drug-resistant cases at this point of elimination may delay the elimination of leprosy. Further research on the physiological characteristics, particularly the parasitic mechanisms, of *M. leprae* will contribute to the development of new therapeutic strategies and drugs and may help realize the goal of “Towards zero leprosy.”

## Abbreviations

*M.leprae*: *Mycobacterium leprae*
*M. tuberculosis*: *Mycobacterium tuberculosis*
WHO: World Health Organization
G2D: Grade-2 disability
LL: lepromatous leprosy
BT: borderline tuberculoid leprosy
BB: mid-borderline leprosy
BL: borderline lepromatous leprosy
TT: tuberculoid leprosy
ENL: erythema nodosum leprosum
PEP: post-exposure prophylaxis
SDR: single dose of rifampicin
PGL-I: phenolic glycolipid I
MDT: multidrug therapy
TLRs: Toll-like receptors
IFN-γ: interferon-γ
TNF-α: tumour necrosis factor-α
iNOS: inducible nitric oxide synthase
NOD: nucleotide-binding oligomerization domain
NLRs: NOD-like receptors
TGF: transforming growth factor
JAG1: protein jagged 1
Hlp: histone-like protein
SNPs: single nucleotide polymorphisms
MAC: membrane attack complex
LAM: lipoarabinomannan
LM: lipomannan
PG: peptidoglycan
AG: arabinogalactan
PDIMs: phthiocerol dimycocerosate
TMM: trehalose mono-mycolate
DAP: diaminopimelic acid
TAG: triacylglycerol
GPAT: glycerol-3-phosphate acyltransferase
LDs: Lipid droplets
ADRP: adipose differentiation-related protein
PPARs: peroxisome proliferator-activated receptors
